# How to evaluate the microcirculation: report of a round table conference

**DOI:** 10.1186/cc6118

**Published:** 2007-09-10

**Authors:** Daniel De Backer, Steven Hollenberg, Christiaan Boerma, Peter Goedhart, Gustavo Büchele, Gustavo Ospina-Tascon, Iwan Dobbe, Can Ince

**Affiliations:** 1 Department of Intensive Care, Erasme University hospital, Université Libre de Bruxelles (ULB), 808 route de Lennik, B-1070 Brussels, Belgium; 2 Sections of Cardiology and Critical Care Medicine, Cooper University Hospital, One Cooper Plazza, Camden 08103, New Jersey, USA; 3 Intensive Care Unit, Medical Centre Leeuwarden, P.O. box 888, 8901 BR Leeuwarden, The Netherlands; 4 Department of Clinical Physiology, Academic Medical Center, University of Amsterdam, Meibergdreef 9, 1105 AZ Amsterdam, The Netherlands

## Abstract

**Introduction:**

Microvascular alterations may play an important role in the development of organ failure in critically ill patients and especially in sepsis. Recent advances in technology have allowed visualization of the microcirculation, but several scoring systems have been used so it is sometimes difficult to compare studies. This paper reports the results of a round table conference that was organized in Amsterdam in November 2006 in order to achieve consensus on image acquisition and analysis.

**Methods:**

The participants convened to discuss the various aspects of image acquisition and the different scores, and a consensus statement was drafted using the Delphi methodology.

**Results:**

The participants identified the following five key points for optimal image acquisition: five sites per organ, avoidance of pressure artifacts, elimination of secretions, adequate focus and contrast adjustment, and recording quality. The scores that can be used to describe numerically the microcirculatory images consist of the following: a measure of vessel density (total and perfused vessel density; two indices of perfusion of the vessels (proportion of perfused vessels and microcirculatory flow index); and a heterogeneity index. In addition, this information should be provided for all vessels and for small vessels (mostly capillaries) identified as smaller than 20 μm. Venular perfusion should be reported as a quality control index, because venules should always be perfused in the absence of pressure artifact. It is anticipated that although this information is currently obtained manually, it is likely that image analysis software will ease analysis in the future.

**Conclusion:**

We proposed that scoring of the microcirculation should include an index of vascular density, assessment of capillary perfusion and a heterogeneity index.

## Introduction

The microcirculation is a commonly neglected entity. Haemodynamic assessment has long been limited to measurements of cardiac output and oxygen delivery, even though microvascular oxygen delivery cannot be predicted from global haemodynamic measurements. Because the microcirculation is the primary site of oxygen and nutrient exchange, therapeutic interventions aimed at increasing organ perfusion should be accompanied by improved microvascular perfusion.

Recent years have witnessed the introduction into clinical practice of devices that allow the microcirculation to be visualized directly. The orthogonal polarization spectral (OPS) [1] and the sidestream dark field (SDF) [2] imaging devices both provide high contrast images of the microvasculature. Both devices are based on the principle that green light illuminates the depth of a tissue (up to 3 mm, according to the manufacturer) and that the scattered green light is absorbed by haemoglobin of red blood cells contained in superficial vessels. Accordingly, both devices allow capillaries and venules to be visualized because these contain red blood cells.

Using these devices, several investigators have reported that the microcirculation is markedly altered in sepsis [3-5], that these alterations are more severe in nonsurvivors than in survivors [3,5], and that persistent microvascular alterations are associated with development of multiple organ failure and death [6]. These alterations typically include decreased vascular density exclusively, caused by decreased capillary density, and decreased perfusion of capillaries. In addition, there can be substantial heterogeneity in microvascular perfusion between areas separated by a few millimetres. In critical illness it has been the sublingual microcirculation that has mostly been studied, and that is the main focus of this report in discussing quantification of the microcirculation. It should be borne in mind, however, that there also can be heterogeneity between different organ systems in critical illness [7].

## Materials and methods

Various scoring systems have been developed by different investigators. In addition, several analytic software packages are under development. Given this high variability in image analysis and given the importance it may have in separating diseased from nondiseased states [3,5,8] and in evaluating the effects of interventions [4,9-13], we organized a round table conference to discuss the various aspects of image acquisition and analysis, and used Delphi methodology to formulate a consensus statement.

### Description of the different scores: principles and limitations

Two scores have been employed until now in clinical practice (Table 1) [3,4].

**Table 1:** Characteristics of the perfusion scores used to assess the microcirculation

	De Backer score [3]	MFI [4]
Variable(s) measured	Total vascular density	Microvascular flow index
	Small vessel density	
	Proportion of perfused vessels (all)	
	Proportion of perfused small vessels (PPV)	
	Perfused vessel density (all)	
	Perfused small vessel density (PVD)	
Main characteristics	Several variables measured, including FCD	Rapid
	Good reproducibility (intra-observer and inter-observer)	Also provides information on type of flow in perfused vessels (sluggish, normal, rapid)
	Continuous variable	Categorical variable
Disadvantages	Score is sensitive to isotropy (change in image size during optical magnification)	Functional capillary density (FCD) not provided

**Figure 1:**
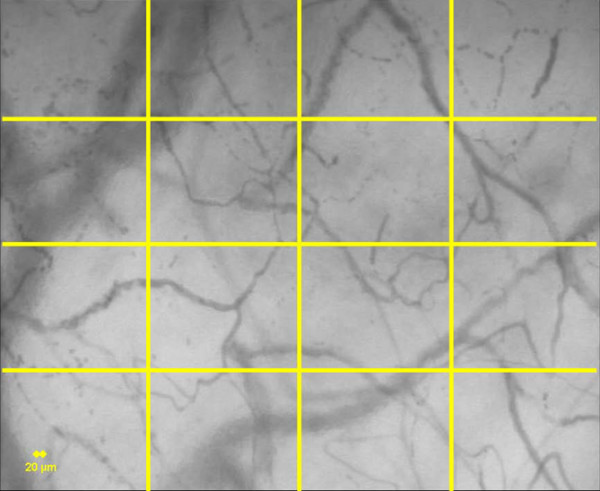
Determination of De Backer's score [3]. Vessel density is calculated as the number of vessels crossing the lines divided by the total length of the lines. Perfusion is then categorized by eye as present (continuous flow for at least 20 s), absent (no flow for at least 20 s) or intermittent (at least 50% of time with no flow). The proportion of perfused vessels (PPV [%]) and perfused vessel density (PVD) are then calculated. A 20 μm cut-off is used to separate small vessels (mostly capillaries) from large vessels (mostly venules).

The first score was developed by De Backer and coworkers [3] and is based on the principle that density of the vessels is proportional to the number of vessels crossing arbitrary lines. In this score, three equidistant horizontal and three equidistant vertical lines are drawn on the screen (Figure 1). Vessel density can be calculated as the number of vessels crossing the lines divided by the total length of the lines. Perfusion can then be categorized by eye as present (continuous flow for at least 20 s), absent (no flow for at least 20 s), or intermittent (at least 50% of the time with no flow). The proportion of perfused vessels (PPV [%]) can be calculated as follows: 100 × (total number of vessels - [no flow + intermittent flow])/total number of vessels. Perfused vessel density (PVD), an estimate of functional capillary density (FCD), can be calculated by multiplying vessel density by the proportion of perfused vessels.

In addition, small vessels (mostly capillaries) were separated from large vessels (mostly venules) using a 20 μm cut-off. The main advantage of this score is that it provides most of the variables involved in organ perfusion, including vascular density and proportion of perfusion. Counting the number of intersections of capillaries with arbitrary grid lines and measurement of total capillary length relative to image surface are similarly reliable measures of FCD [14]. Reproducibility of this semiquantitative score is excellent, with an intra-observer variability ranging between 2.5% and 4.7% for vessel density and between 0.9% and 4.5% for vessel perfusion [3]. The inter-observer variability is slightly higher (at between 3.0% and 6.2% and between 4.1% and 10%, respectively). Although the images are stored using random numbers, they are analyzed in batches of images by a single investigator so that the intra-observer variability applies when effects of interventions are investigated. To prevent drift in analysis, images are regularly reviewed by several investigators. A disadvantage of the score is that it takes no account of the velocity of red blood cells, provided that flow is continuous. In addition, the length of the line can vary according to the magnification, which may be a problem when post-acquisition manipulation of the image is performed (software that provides image stabilization may resize the image so that the final image may have a magnification different from that of the original).

**Figure 2:**
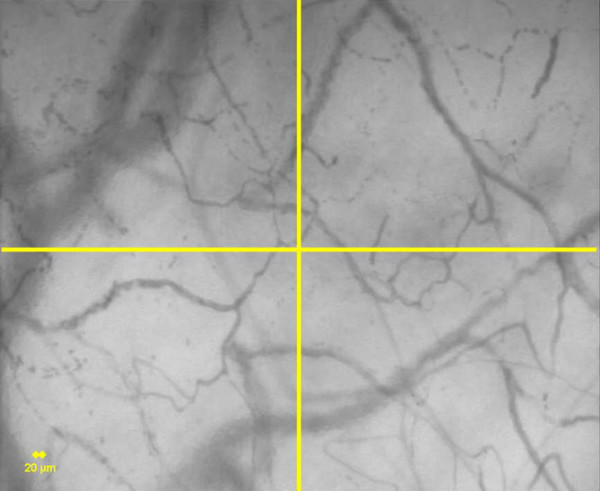
Determination of mean flow index (MFI) score [15]. The image is divided into four quadrants and the predominant type of flow (absent = 0, intermittent = 1, sluggish = 2, and normal = 3) is assessed in each quadrant. The MFI score represents the averaged values of the four. A 20 μm cut-off is used to separate small vessels (mostly capillaries) from large vessels (mostly venules).

The second score is the microvascular flow index (MFI) score [4,5,15]. This score is based on determination of the predominant type of flow in four quadrants (Figure 2). Flow is characterized as absent (0), intermittent (1), sluggish (2), or normal (3). The values of the four quadrants are averaged. The main advantage of this score is that it is relatively easy to measure. It also takes into account the fact that flow can be continuous but very slow (sluggish). The reproducibility of the test was recently investigated by Boerma and coworkers [15]. These authors reported an intra-observer agreement of 85% (Kappa score 0.78) and inter-observer agreement of 90% (Kappa score 0.85). A similar inter-observer reproducibility was recently reported by Trzeciak and colleagues [5] (Kappa score 0.77). The main disadvantage is that it does not provide information about FCD. Accordingly one cannot exclude that an intervention improved flow in the vessels that are visualized but that the number of perfused vessels decreased, which might result in an impaired microvascular perfusion. In addition, the score is ordinal and thus discontinuous; it ranges from 0 to 3, and a change from 0 to 1 may not have the same implications for tissue perfusion as a change from 2 to 3, which may complicate the interpretation of the effects of therapeutic interventions.

The two scores can be combined, as was recently done by Trzeciak and coworkers [5] who used MFI to evaluate the type of flow and the six lines (three horizontal, three vertical) technique to evaluate vessel density. In addition, those authors developed an interesting index to assess flow heterogeneity between the different areas investigated. This heterogeneity index was calculated as the highest site flow velocity minus the lowest site flow velocity, divided by the mean flow velocity of all sublingual sites.

### Theoretical and practical considerations

In analyzing microvascular images there are trade-offs to be made, and several theoretical and practical considerations may influence these choices. The subtler the changes one is attempting to detect, the greater is the expertise required in image analysis. Detection of large changes is easier but adds less to more readily measurable parameters.

Perhaps most crucial is the element of time required to perform the analysis. Detecting subtler abnormalities and increasing the precision of the measurements inevitably increases the time required to make the determination. In addition to making the analysis more tedious, the longer the analysis takes the less applicable it may be to the clinical situation, because clinical status of patients evolves over time.

Microvascular assessments are most likely to add incremental value in patient management to the extent that results can be applied expeditiously at the bedside. The immediacy of these results must be traded off against considerations of accuracy and reproducibility.

The measured variables should thus be relatively easy to measure and should have pathophysiological implications.

## Results and discussion

### Consensus regarding image acquisition

The five consensual key points for image acquisition are summarized in Table 2.

**Table 2:** The five key points for optimal image acquisition

Point	Details
1	Five sites per organ
2	Avoidance of pressure artefacts
3	Elimination of secretions
4	Adequate focus and contrast adjustment
5	High quality recording

#### Number of sites in a specific organ

Given the intrinsic variability of the microcirculation [3,5], several sites of the organ of interest should be averaged. Ideally five sites should be examined, but at image analysis the quality of some images may be less than initially estimated and these should be discarded. Accordingly, we concluded that at least three sites that can be reliably evaluated per patient, and if possible five sites, should be obtained at each evaluation.

#### Adequate choice of optical magnification

One may wish to increase optic magnification in order to enhance visualization of some structures (white blood cells). On the other hand, the increased microscopic precision limits the field of interest to a narrower window, which may be problematic, considering the heterogeneity of the microcirculation. In addition, movement artifacts will be magnified. Accordingly, we recommend use of 5× objectives for human sublingual microcirculation with OPS and SDF devices. In small animals, 10× objectives should be used.

#### Pressure artifacts should be eliminated

Capillaries and venules are collapsible; accordingly, these vessels may be very sensitive to pressure applied to the organ. Because the microcirculation is just below the microscope, excess pressure applied to the area may collapse the microcirculation, and the investigation of the microcirculation can become unreliable in these conditions. This can result in decreased flow in large venules (venules >30 μm), which may become sluggish, absent, or alternate, or there may even be backflow. Importantly, pressure may be only focal, when the pressure is not applied globally to the preparation but only to one side. Of note, pressure artifacts can also be observed during compression of the tongue (for instance, by the investigators' finger, in an attempt to stabilize the tongue) or during contraction of tongue muscles. Interestingly, all authors have reported that venular perfusion is always preserved, whatever the severity of alteration in smaller vessels [3,8]. Observation of an altered large venular blood flow is thus suggestive of a pressure artifact. To prevent applying pressure to the area, it is recommended that the microscope be pulled back gently until contact is lost and then to advance the probe again slowly to the point at which contact is regained. These aspects are summarized in the operational procedure proposed by Trzeciak and coworkers [5].

#### Minimal technical setup

Several technical issues should be addressed to ensure adequate image acquisition and further analysis. Video images are usually immediately captured on a computer using a dedicated videocard, and the images should be stored at full size as DV-AVI files to allow computerized frame-by-frame image analysis and use for educational purposes. We recommend limiting recording time to 20 s because it may be difficult to maintain a clear and steady image for a longer period. In addition, longer clips should be divided for further analysis, especially if analysis is performed using software. Clips of 20 s duration are already very large (50 to 100 MB), and the need for adequate storage should be anticipated. To enhance image focusing, large external monitors should be used instead of the LCD screen of the computer. Videotaping the image (and later digitalization of the images) can also be performed if needed, but high-quality digital videotape recording and appropriate labelling of the video strips are necessary. VHS video recording or DVD recording where MPEG compression is used should be avoided because these result in loss of resolution.

### Consensus regarding image analysis

Several determinations should be made during image analysis. First, capillaries should be differentiated from venules, because capillaries contribute predominantly to organ perfusion. Second, perfusion should be estimated. The perfused capillary density is probably the most important variable to determine because it is factor with the greatest influence on perfusion. In addition, it is also important to determine perfusion heterogeneity, which is a crucial determinant of extraction capabilities of the tissue [16-18].

The usefulness of determining the speed of blood in the vessels is uncertain. Homogeneity of perfusion is more important than blood velocity in assuring tissue oxygenation, because cells are able to regulate oxygen extraction in the presence of variable flow. Accordingly, a homogenous low flow (sluggish) may be better tolerated than a heterogenous flow, even when total blood flow is lower [19]. The consequences of very high blood flow are not well known. From a theoretical point of view, very high flow may induce shear-stress lesions to the capillary wall, promoting further microvascular lesions, and may impair oxygen offloading. However, the importance of these phenomena has not been demonstrated in the clinical setting. For this reason, very high flow is not taken into account in the different scores.

#### Choice of diameter

It is difficult to separate venules from capillaries. Usually, these vessels are delineated according to their diameter and a cut-off value of 20 μm is used to differentiate capillaries from venules. However, the size of capillaries and venules can be affected by various factors, so this limit can fluctuate. Analyses of larger vessels are of limited interest except as a quality control measure to ensure that no excessive pressure is applied to the tissue. In larger venules, rolling and adherent leucocytes can be observed, but this requires higher magnification and different analytical methods.

#### Quadrants

Separation of the screen into quadrants (or using equidistant lines) is mandatory when analysis is done by eye. Indeed, it is very difficult to count vessels over the entire screen because the eye may be attracted by specific regions of interest. However, the altered microcirculation is usually heterogeneous, and it is thus important to have a full overview of the image. To obtain a comprehensive measure of the perfusion characteristics, it is advisable to measure both the MFI, and the PVD and PPV. The image is divided into four quadrants and flow is assessed in each quadrant to measure the MFI [15]. Three horizontal and three vertical lines are drawn on the screen, and perfusion of each vessel at an intersection with lines drawn on the screen is determined to measure the PVD and PPV [3] of the image. This type of comprehensive analysis (for example, MFI, PPV and PVD) helps to generate a picture of perfusion and perfusion heterogeneity in representative types of vessels, avoiding oversimplification. (Additional files 1 to 4). Drawing quadrants or lines may be obsolete if perfusion of all vessels can be detected by software analyses.

#### Measured variables: FCD

FCD, estimated as PVD, can be calculated either as the number of perfused vessels that cross three horizontal and three vertical lines, divided by total length of lines (as in the report by De Backer and coworkers [3]) or as total length of perfused vessels divided by total surface of area (with appropriate software) [20].

**Figure 3:**
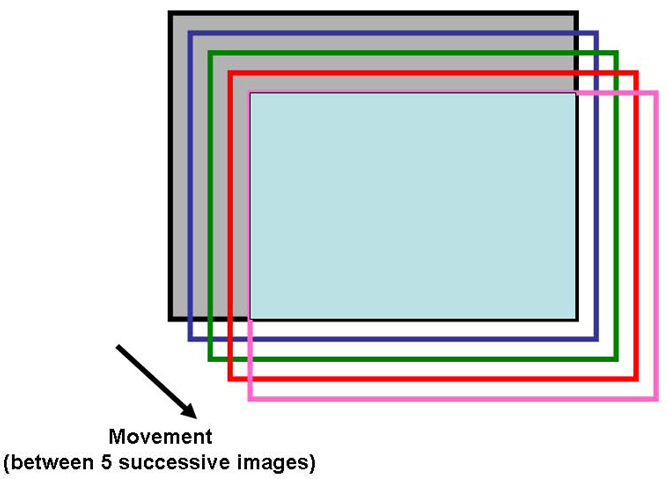
Change in image size during software stabilization. When movements occur, software can re-centre the image using easily recognized structures. However, peripheral parts of the images, not seen on successive images, will be lost so that the final area will be smaller than the original one. The size of the original image is represented by the light grey rectangle, and the final one by the light blue rectangle.

Perfused vessels are defined as total number of vessels - (no flow + intermittent vessels). These may be calculated for each type of vessel. Problems may be encountered when the vessel diameter is incorrectly identified and with looping vessels that may be counted twice. Calculation should be made only in images that have not been manipulated. Software can be helpful in stabilizing the image, but this procedure implies some size reduction (Figure 3). The total length of the lines will be affected by this procedure.

To calculate the total length of the lines, we must know exactly the size of the image projected on the screen. The US National Television Systems Committee (NTSC) standard and the phase alternating line (PAL) and sequential colour with memory (SECAM) standards use different displays that may affect the presentation on the screen (720 × 576 pixels for DV-PAL and 720 × 480 pixels for DV-NTSC). The resulting differences in area should be taken into account when using a scoring method worldwide. The optical field of view of SDF imaging with 5× objectives is approximately 0.94 mm × 0.75 mm. A slight difference between the magnifications between OPS and SDF explain small differences in image size. In PAL/SECAM standard the OPS system gives an image size of 1.54 mm × 1.15 mm (1.54 mm × 0.96 mm in NTSC), and the SDF gives an image size of 0.98 × 0.73 mm (0.98 mm × 0.60 mm in NTSC). Of note, the length and width of both systems can slightly vary during focusing because both OPS and SDF devices focus by moving the camera closer or further away from the tissue, altering the magnification. Usually, this effect is quite limited but it can be as large as 10% when the full range of focus (0 to 1 mm depth [1]) is explored.

#### Measured variables: flow index

The ideal software, we propose, should automatically recognize all blood vessels and measure their diameters and blood flow in each individual vessel of the investigated field. This is not currently available. Semi-quantitative analysis should therefore be used; such analysis has been proven to be able to distinguish between health and disease [3,5,8].

Our consensus is that all three indices discussed above (PVD, PPV and MFI) should be measured to describe comprehensively the functional perfusion of the microcirculation. Looking at PPV allows no distinction to be made between normal, sluggish and hyperdynamic flows, but it provides information on flow heterogeneity within the image. PVD provides an accurate estimate of FCD.

In addition to making the distinction between perfusion and nonperfusion, the MFI score differentiates between the different types of continuous flows (sluggish, normal and high flow). In conditions where flow is homogeneous, the MFI score can thus provide additional information. However, with this method the capillary density, and thus FCD, is not estimated. Hence, the proportion of perfusion should be used in heterogeneous situations, whereas MFI should be preferred in more homogenous conditions because it takes into account the difference between sluggish and continuous flows.

#### Measured variables: heterogeneity index

The microcirculation is heterogeneous in many disease states, and for this reason it has been proposed that several areas be averaged. The heterogeneity can be quantified. Initially, the coefficient of variability was used [3]. More recently, Trzeciak and coworkers [5] proposed another heterogeneity index, which involves evaluating three to five sites and measuring the MFI in the quadrants, taking the difference between highest MFI minus the lowest site MFI divided by the mean flow velocity of all sublingual sites at a single time point. This heterogeneity index has the advantage of taking into account extreme deviations, whereas the coefficient of variation evaluates all deviations from the mean. From a pathophysiological point of view, the heterogeneity is a key determinant of the shunted fraction, often seen in distributive shock. For this reason, taking into account the extreme deviations is more representative.

#### What should be included in a report of the analysis of the microcirculation?

An analysis of the microcirculation (Table 3) should be reported for both total and small (<20 μm) vessels. The consensus is to report PVD, PPV and MFI to describe the functional perfusion of the microcirculatory image. The heterogeneity index (calculated as the difference between extreme values of either MFI or PPV between the three to five recordings of the organ divided by its mean value) is needed to describe the heterogeneity of perfusion in the microcirculatory area under observation.

**Table 3:** The ideal analysis report

Component of report	Measure (if applicable)	Details (if applicable)
Vessel density	Total vessel density	
	Perfused vessel density (PVD)	All (n/mm)^a ^Small vessels (n/mm)^a^
Perfusion indices	Proportion of perfused vessels (PPV [%])	All Large vessels Small vessels
	Microvascular flow index (MFI)	All Large vessels Small vessels
Heterogeneity index (%)		

^a^Vessel density is expressed as mm/mm^2 ^if software is used to draw vessel length (and calculated as perfused vessel length/investigated area.

#### Interpretation of the score

The interpretation of these variables may sometimes be difficult, especially when discordant changes between the different indices occur during interventions.

Tissue perfusion is dependent on FCD (reflected by PVD) and blood velocity (reflected by MFI). As discussed above, vascular density is probably more important than blood velocity in determining tissue perfusion, because oxygen extraction can compensate for a decreased flow. Shunt fraction, a key determinant of oxygen extraction capabilities [16,21,22], is reflected by blood flow heterogeneity in the investigated area by PPV and between the different areas of the investigated organ by the heterogeneity index.

#### Software

Several software packages have been developed, allowing FCD calculation or reliable blood flow measurements in individual vessels. The CapImage software (Dr Zeintl software Engineering, Heidelberg, Germany) has been developed for intravital microscopy [23] but can also be used for the analysis of OPS and SDF images [24,25]. This software is validated for blood flow measurements in straight vessels segments only. The CapiScope software (KK Technology; Honiton, UK) has been developed for analysis of OPS images. It measures FCD, and vessel diameter and velocity. It reliably measures blood flow in individual vessels. Very stable images, without any movement artifact, should be used with these two software packages because they do not provide image stabilization. Recently, the MAS analysis system (MicroVision Medical, Amsterdam, The Netherlands) was developed. It includes a stabilization image processing, a calculation of FCD and measurements of blood flow in individual vessels. Unfortunately, these packages still require much user intervention to identify the vessels of interest. In addition, flow cannot be calculated automatically and simultaneously in multiple vessels, so that blood flow distribution histograms can not readily be obtained. In addition, it is particularly difficult to measure blood flow in capillaries, which constitute the main area of interest. Blood flow measurement is calculated as cross-sectional area (based on measurement of vessel diameter) times blood velocity. The error in determining the flow is especially large with errors in measurement of vessel diameter, since it is the square of the diameter that is used in cross-sectional area (πD^2^/4) calculation. Determination of vessel diameter is difficult in small capillaries, and consequently the relative error in measurements may be greatest in small vessels. Vessels are visualized because they contain red blood cells but the vessel wall is not visualized. In most vessels, multiple red blood cells flow side by side, allowing easy identification of vessel diameter. This is more complicated in small capillaries, especially when red blood cells are separated by plasma gaps. Software with time averaging of sequential frames and better imaging modalities may improve the accuracy of these measurements.

One may anticipate that in the future FCD measurement will be mechanized. Although FCD may be obtained automatically, this process is likely to require some human validation, ideally by clicking away vessels that do not appear to be perfused. The human eye can easily draw a vessel when red blood cells are separated by large plasma gap, whereas this is will probably continue to be a limitation of software in the short term.

All variables should be separated according to vessel size using a cut-off of 20 μm. Histogram of vessel diameter and vessel flow would provide not only mean values but also identify variability in the measurements.

Vessel flow measurements require a moving feature (isolated red blood cell or white blood cell) to be visible in at least three consecutive movie frames. The highest computer-aided measurable velocity is physically restricted by the video frame rate (30 frames/s for NTSC and 25 frames/s for PAL and SECAM) and by the length of the vessel part where the flow is assessed. Faster cameras with higher frame rates could overcome this physical limitation. This is important because with current conventional cameras the flow in fastest flowing vessels can not be calculated [26].

Stabilization processes incorporated in software packages are very helpful in improving image readability and computerized analysis. However, problems of isotropy (see above) are encountered when FCD is determined semi-quantitatively using the six lines methods. Independently of the analytical method used, some information will be lost. Indeed, peripheral parts of the images, not seen on successive images when movements occur, will be lost during the stabilization process (Figure 3). As a result, the final image is smaller than the original one, but the software displays this transformed image at the same size as the original image, altering its magnification. Ideally, the percentage of reduction from the original size should be provided by the stabilization software, but this information is not currently provided.

### Specificities of microvascular networks

All of these methods have been developed in the sublingual area, where vessels project in random directions. Accordingly, orientation of the camera, and hence the lines, have no effect on calculation of FCD. In other types of vascular structures, it may be appropriate to use different types of analyses. When vessels flow in parallel, lines perpendicular to the orientation of the vessels should be used. For microvilli and crypts, one may count the perfused units compared with the total number of visualized units [7,15,27].

## Conclusion

The scoring of the microcirculation should include an index of vascular density, assessment of capillary perfusion and a heterogeneity index. The consensus advises reporting of PVD, PPV, MFI and heterogeneity index, in order to describe the functional perfusion of the microcirculation.

Additional files 1 to 4 provide four representative videos analyzed according to our consensus proposition, based on De Backer's score [3] and MFI score [15] (heterogeneity index is not determined on isolated images).

## Key messages

• Analysis of the microcirculation can be reliably achieved using semi-quantitative scores.

• Scoring should include measurement of perfused capillary density and evaluation of heterogeneity. We propose that PVD, PPV and MFI should be measured. Heterogeneity index should be calculated.

• Image acquisition should include at least three good quality sequences of 20 s each. Absence of perfusion in large veins suggests a pressure artifact.

## Abbreviations

FCD = functional capillary density; MFI = microcirculatory flow index; NTSC = National Television Systems Committee; OPS = orthogonal polarization spectral; PAL = phase alternating line; PPV = proportion of perfused vessels; PVD = perfused vessel density; SDF = sidestream dark field; SECAM = sequential colour with memory.

## Competing interests

DDB, SH, CB, PG, GB, GOT and ID had no conflict of interest in relation to the current work; CI is Chief Scientific Officer of MicroVision (a university-based company manufacturing SDF devices).

## Authors' contributions

All authors actively participated in the debates during the round table conference. The drafts of the manuscript were written by DDB and all authors contributed to writing of the manuscript, which was circulated among each of them.

## Supplementary Material

Additional file 1 A video clip file showing normal microcirculation in a healthy volunteer. Thirty-eight small vessels (including one with absent flow and none with intermittent flow) and 23 large vessels (all perfused) are visualized. MFIs for each quadrant determined clockwise from the left upper one are 3, 3, 3 and 3. Accordingly, PPV is 95%, PVD is 7.1/mm and MFI is 3. Click here for file

Additional file 2 A video clip file showing altered microcirculation in a patient with severe sepsis. Forty-nine small vessels (including four with absent flow and eight with intermittent flow) and 19 large vessels (all perfused) are visualized. MFIs for each quadrant determined clockwise from the left upper one are 3, 0, 3 and 3. Accordingly, PPV is 61%, PVD is 5.9/mm and MFI is 2.25. Click here for file

Additional file 3 A video clip file showing altered microcirculation in a patient with severe sepsis. Thirty-six small vessels (including one with absent flow and four with intermittent flow) and 25 large vessels (all perfused) are visualized. MFIs for each quadrant determined clockwise from the left upper one are 3, 3, 3 and 3. Accordingly, PPV is 76%, PVD is 5.4/mm and MFI is 3. Click here for file

Additional file 4 A video clip file showing severely altered microcirculation in a patient with sever sepsis. Forty-six small vessels (including 20 with absent flow and 14 with intermittent flow) and 15 large vessels (all perfused) are visualized. MFIs for each quadrant determined clockwise from the left upper one are 0, 0, 3 and 0. Accordingly, PPV is 15%, PVD is 1.4/mm and MFI is 0.75. Click here for file
